# The quality of life and inequalities in health services for epilepsy treatment among patience in the urban cities of Sudan

**DOI:** 10.11604/pamj.2019.33.10.15440

**Published:** 2019-05-07

**Authors:** Muwada Bashir Awad Bashir, Samuel Nambile Cumber

**Affiliations:** 1 Section for Epidemiology and Social Medicine, Department of Public Health, Institute of Medicine (EPSO), The Sahlgrenska Academy at University of Gothenburg, Gothenburg, Sweden; 2 Discipline of Medicine and Surgery, Faculty of Medicine University of Khartoum, Sudan; 3 Faculty of Health Sciences, University of the Free State, Bloemfontein, South Africa; 4 School of Health Systems and Public Health Faculty of Health Sciences, University of Pretoria Private Bag X323, Gezina, Pretoria, 0001, Pretoria, South Africa

**Keywords:** Epilepsy, patients, quality of life, inequalities, Sudan

## Abstract

Epilepsy in Sudan accounts for 1.6 annual mortality rates and 238.7 disability adjusted life years per 100 000. These figures are higher among females; children and young adults. It is associated with notable stigma and social burdens. Patients of epilepsy are subjected to various forms of social discrimination that affect their quality of life. They are isolated, neglected and deprived of their education and employments rights and not able to achieve normal social and family life. Aiming at highlighting social implications of epilepsy among Sudanese patients, this study found that social encumbrances due to epilepsy in Sudan are more prevalent among highly vulnerable groups like women, children and poor populations living in remote areas. Lack of trained medical personnel in neurology and the medical equipment's required for proper diagnosis and treatment of epilepsy in Sudan are key reasons aggravating social and health burden of epilepsy both among patients and their caregivers.

## To the editors of the Pan African Medical Journal

Epilepsy is a chronic disease characterized by episodes of seizures that may vary from brief undetectable to long periods of vigorous shaking and could be accompanied by physical injuries like bone fractures [1]. Eighty percent of epilepsy patients live in in low- and middle-income countries and five hundred million of them are closely involved as family and colleagues. Although three fourths of the epilepsy in the word is treatable, yet 85% do not get any treatment at all. With these figures speaking that it is one of the most common global health challenges as a non-communicable disease, contributing to massive social and economic burdens on societies, Stigmatization, social discrimination and inadequate health services are major problems that epileptic patients and their families confront in their daily life, which are of direct implication with factors like culture, education and local costumes and traditions [2]. In Sudan epilepsy accounts for 1.6 annual mortality rates with 238.7 disability adjusted life years per 100 000. They are higher among females, children and young adults [3]. In a recent study in 2016 Prevalence of epilepsy among children in Khartoum state was estimated to be 4 per 1000, males were more affected than females with ratio of 1.5:1 and higher statistics reported in rural areas than affluent ones. Such figures are significantly higher than previously reported prevalence of .9 per 1000 in 1983 [4]. Up to date data about incidences and prevalence of epilepsy in Sudan are very deficient. Most of the currently available data are retrieved from cross sectional and small population-based studies [2]. The aim of this letter is to review the quality of life of patients with epilepsy in Sudan in relevance to stigmatization and social implications affecting the disease outcome. To highlight inequalities in health services in epilepsy treatment in Sudan. In this short letter, 13 data base were used during the search for articles/information. The data base used were; EBSCO, PubMed, ECO, LIBRIS, Web of Science, ArticleFirst, DIVA, PsycINFO, Africa Bibliography, African Journal On-Line, Education Resources Information Centre, Google Scholar and SwePub. The above 13 data bases were used to search for articles, journals and reports. From the literature search, 27 citations were found, using keywords such as Epilepsy; Patients; Quality of life; Inequalities; Sudan. Only 9 articles met the interior criteria and were those 9 used to answer the specific objectives for this letter.

Epilepsy in Sudan is associated with notable stigma, social burden and generally affects the quality of life of patients [5]. Subjected to various forms of social discrimination that affect their quality of life, patients are deprived of their education and employments rights because of their diseases. For example, females cannot marry and have children or might be abandoned by their families because of their sickness. Also, in rural areas where the disease is perceived to be caused by devil forces and superstition, patients and their families are treated as cursed people; they are neglected, isolated and treated with inferiority [2, 5]. Studies conducted to assess epilepsy related stigma and quality of life in Sudan revealed that at least half of the patients had positive felt stigma [5]. When compared to their controls, a significant lower quality of life was reported among patients, while for children, striking figures showed that more than have of them were not attending school because of their illness, one third had learning disabilities and 10% with motor disability. Care givers were mostly parents or siblings 66.2%, others were spouses or own children of the patients 15.2%. Majority of them were women 42% who are married, in low skilled occupation and barely had secondary school education [6]. Most of the studies reported better quality outcome when care givers are males, age is less than forty, better occupational status and living in urban setting. In another context patient's relations with their families and friends were not found to be negatively affected by the disease [6]. However, it impaired their education, employment and daily life activities and social aspects like travel and sport [7]. The major concerns for epileptic patients are the possibilities of self-injury or suffocation especially with the treatment altering behavior and consciousness effect [7]. Others were about children foreseen probability of having mood change bouts, difficulty in socialization, forming friendships, marriage and family build up, while families burdening concerns were about using public transport and explaining the patient's problem to others. Mothers reported difficulties with giving other siblings enough attention with the most dedicated to their disease's child and problems with limiting family going out, work and entertainments activities [6, 7].

Epilepsy also affects the health services, inequality and the treatment gap. A simple definition of treatment gap in epilepsy is the number of people with a condition or disease who need treatment for it but do not get it. In the developing world, this gab is either arising from difficulty in accessing biomedical services or lack of adherence to anti-epileptic drugs [2]. In Sudan there have not been specific studies conducted for assessing heath care services and treatment gabs in epilepsy specifically. Lack of trained medical personnel in neurology. Basic, advanced diagnostic and medical equipment's required for diagnosis and treatment are limited in both urban and rural areas in Sudan and proper specialized neurological services [8, 9]. In some peripheral states no neurology specialist are there to provide such specialized services and diagnostic facilities like magnetic resonance imaging (MRI) and computerized axial tomography (CT) are also not available. Electroencephalogram (EEG) machines are available only in two psychiatric hospitals in the capital city where most of epileptic patients receive services [8]. Another studies have demonstrated the significant figures of Sudanese patients who seek for alternative care like traditional healing when it comes to epilepsy [9]. A huge disparity in levels of satisfaction, social impairment and quality of life has been showed between rural and urban areas, males and females and children and adults patients. The negative consequences of the diseases were more prevalent among females, children and poor populations living in rural areas [6, 7]. Considering the socio-cultural paradigm in Sudan, these findings are explainable by the fact that the culture in Sudan is being influence by Sufism and Islamic religion, the thing highly mitigating the issue of family and social acceptance and support of patients. In Islam mentally ill people are perceived as blessed ones who evoke goodness to their families and surroundings but in the same context such high dependence on family aggravates the psycho-social and economic burdens they tackle as care givers [10]. Furthermore, such notion justifies the highly reported numbers of patients seek traditional healing care instead of medical treatment as it improves the social and cultural acceptability of their disease. The highest figures of utilizing traditional healing reported among rural and poor communities, in turn, reflect medical services access and utilization barriers perceived among rural populations. In line with finding stating that epilepsy treatment gab disparities are 75% more among lower income classes, rural populations and vulnerable social groups, reported in other studies including countries like Uganda, Ethiopia and Tanzania [2, 10].

In the other hand the marked impact in the education, employment and daily life activities is attributable to lack of education and awareness raising programs about epilepsy any of which Sudan lacks to help incorporate these patients into their social roles. In addition to health services centralization in metropolitan areas, depriving remote areas inhabitants from their goods. The findings of Women and children marked impediment by the diseases are consistent with similar results reporting lower parameters for quality of life in same social groups in Nigeria and Egypt [10]. They reflects a low level of women empowerment and age and gender sensitive medical care directions in Sudan. Such inequalities are explainable by absence of structured specialized national programs for overseeing epileptic medical, social and welfare services and the huge deficiency in research actions. This, in turn, could be attributed to the high cost of quality studies needed for addressing the problem and the stalling of active programs of monitoring and surveillances for the progress in the global NCD targets in Sudan. To conclude, epilepsy causes major health and social burden in Sudan affecting mostly vulnerable social groups. Factors as Cultural and social acceptability, awareness, stigmatization and inadequacy of health services to address patients and their family needs are aggravating both patients and care givers encumbrances in term of health care accessibility and dealing with the disease. Wide based quantitative and qualitative studies addressing socio cultural, human rights implications and disparities of epilepsy health care among different socio-economic statuses in Sudan are highly needed. Structured and specialized program must be established both in national and state level for raising public awareness and modifying socio-cultural attributes of the diseases. Health care and social welfare services for epilepsy patients need to be well structured with considerable age and gender sensitivity to meet the health requirements for vulnerable social groups in Sudan and Traditional health services for epilepsy should be oversee and organized towards maximum benefit achievement by the patients through integration, coordination and organization with medical care system.

## Competing interests

The authors declare no competing interests.
